# Urinary excretion and metabolism of procyanidins in pigs

**DOI:** 10.1002/mnfr.201100471

**Published:** 2012-04-12

**Authors:** Sebastian Rzeppa, Katharina Bittner, Susanne Döll, Sven Dänicke, Hans-Ulrich Humpf

**Affiliations:** 1 Institute of Food Chemistry, Westfälische Wilhelms-Universität MünsterGermany; 2 Institute of Animal Nutrition, Friedrich-Loeffler-Institute (FLI), Federal Research Institute for Animal HealthBraunschweig, Germany

**Keywords:** Flavanoids, HPLC-MS, MS, Metabolism, Pigs, Procyanidins, Excretion

## Abstract

**Scope:**

Aim of this study was to investigate urinary excretion and metabolism of procyanidins a group of secondary plant metabolites with many beneficial health effects described in literature.

**Methods and results:**

To investigate the metabolism of procyanidins in the absence of flavan-3-ols, centrifugal partition chromatography was used for their reduction in a grape seed extract to a level of almost zero. After administration of the monomer reduced grape seed extract (mredGSE) containing procyanidins B1, B2, B3, B4, C1 to pigs flavan-3-ols, their methyl derivatives, dimeric and trimeric procyanidins were determined in urine by high-performance liquid chromatography tandem mass spectrometry (HPLC-MS/MS). Maximal concentrations of procyanidins 6 h after administration vary from 5 to 30 ng/mg creatinine. Total excretion of flavan-3-ols and their methyl derivatives indicates an increasing trend for pigs given mredGSE in comparison to pigs of the control group. Flavan-3-ols were conjugated and methylated to a great extent in comparison to dimeric and trimeric procyanidins. In the case of low molecular weight metabolites, an increasing trend was observed for hippuric acid, not for phenolic acids.

**Conclusions:**

Ratios of total excretion of procyanidins to administrated amounts between 0.004% (C1) and 0.019% (B4) suggest a poor urinary excretion by pigs. A transfer of these results to humans is possible due to their similar gastrointestinal tract.

## 1 Introduction

Proanthocyanidins as secondary plant metabolites occur in a large number of foods and their concentrations are relatively high in comparison to other polyphenols. The most important sources for humans are fruits (apples, grapes, berries, plums), beverages (tea, cocoa, wine), cereals (barley, sorghum), nuts, and chocolate [1, 2]. The daily intake level is not precisely known because of the lack of reliable values for proanthocyanidins in food. Estimated consumption given by Gu *et al.* is 53.7 mg proanthocyanidins/day [2004]. Many beneficial health effects are described for proanthocyanidins in literature, e.g. antioxidant and radical-scaving activities [3, 4] and a growth inhibition of human cancer cell lines [5]. In relationship to the so called “French paradox,” a reduction of the incidence of cardiovascular diseases is possible [6], e.g. in the case of cocoa procyanidins [7].

**Figure 1:**
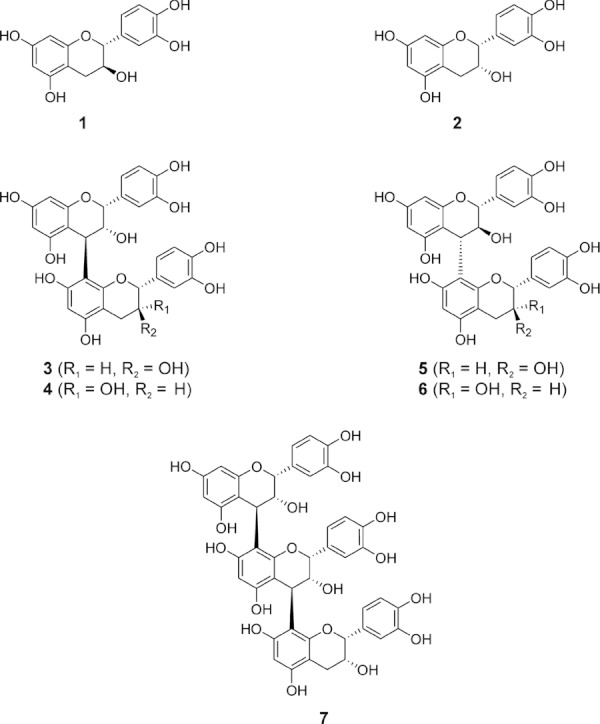
Structures of flavan-3-ols and procyanidins as main constituents of grape seed extract: (+)-catechin (1), (–)-epicatechin (2), procyanidin B1 (3), B2 (4), B3 (5), B4 (6), and C1 (7).

The most common proanthocyanidins are procyanidins that are oligomers and polymers of the flavan-3-ols catechin (CT) and epicatechin (EC) (see Fig. 1). B-type procyanidins are linked by a C4–C8 bond, but C4–C6 linkages also occur. A-type procyanidins bear in addition to the C–C linkage, an ether bond between C2 and O7 or C2 and O5 [8].

However, the knowledge concerning bioavailability and metabolism is rather limited. A possible absorption of procyanidins by the human body is discussed controversially in literature. An absorption/excretion of procyanidins *in vivo* by the identification of these substances in plasma and/or urine was described for rats [9, 10, 11, 12, 13]. For example, Shoji *et al.* identified oligomeric procyanidins in rat plasma after administration of apple condensed tannins [2006]. The detection of dimeric procyanidins in human plasma after intake of cocoa or grape seed extract (GSE) was also possible [14, 15]. After consumption of cocoa, Urpi-Sado *et al.* identified procyanidin B2 in human urine [16]. On the other hand, results of other studies using rats point against an *in vivo* absorption of procyanidins [17, 18]. Donovan *et al.* could detect no dimeric procyanidin B3 after administration of this compound to rats [2002]. Besides the procyanidins itself, it is interesting to have a deeper look into possible degradation products that are formed during the passage of the gastrointestinal tract. A possible degradation of oligomeric and polymeric procyanidins into smaller units, especially flavan-3-ols, formed by gastric juice, is discussed controversially in literature [19, 20, 21]. Spencer *et al.* observed a degradation of oligomeric procyanidins (trimer to hexamer) into flavan-3-ols and dimers in simulated gastric juice [2000]. Flavan-3-ols are bioavailable after intake of food samples containing CT or EC, such as in the case of chocolate [22, 23]. A possible degradation into flavan-3-ols can be an explanation for health effect of procyanidins. Furthermore, the formation of low molecular weight degradation products such as phenolic acids by the intestinal microbiota has been described in literature. The formation of these compounds is described for *in vitro* experiments [24, 25, 26, 27] such as the pig cecum model [27] as well as for *in vivo* experiments [28, 29, 30]. Low molecular weight phenolic compounds can be absorbed much easier and may contribute to the beneficial health effects [31].

Aim of this study was to investigate the urinary excretion and metabolism of procyanidins selectively and not in the presence of flavan-3-ols. This study design is in contrast to that of most other studies that used food samples or commercial plant extracts with a more or less high content of flavan-3-ols. Therefore, a centrifugal partition chromatography (CPC) method was developed that allows the reduction of flavan-3-ols in a GSE to a level of almost zero. This monomer reduced grape seed extract (mredGSE) contains the dimeric procyanidins B1, B2, B3, and B4 as well as the trimeric procyanidin C1 as main constituents beside further oligomeric and polymeric procyanidins (see Fig. 1). In cooperation with the Institute of Animal Nutrition, Friedrich-Loeffler-Institute (FLI), this extract was orally administrated to pigs in a kinetic study. Pigs have a gastrointestinal tract that is very similar to humans. For example, the composition of the intestinal microbiota is very similar to humans [32]. Analysis of flavan-3-ols, procyanidins, and their conjugated compounds in urine was performed with high-performance liquid chromatography-tandem mass spectrometry (HPLC-MS/MS) in combination with a new solid phase extraction method using Sephadex LH20. Glucuronides were quantified indirectly by degradation with β-glucuronidase and confirmed by HPLC coupled with fourier transform mass spectrometry (HPLC-FTMS). Low molecular weight metabolites of procyanidins namely phenolic acids were investigated by gas chromatography-mass spectrometry (GC-MS) and high-performance liquid chromatography-diode-array detection (HPLC-DAD).

## 2 Materials and methods

### 2.1 Chemicals

Solvents used for sample extraction and chromatography were obtained from VWR (Darmstadt, Germany), Sigma-Aldrich (Steinheim, Germany), and Carl Roth (Karlsruhe, Germany). Water was purified with a MilliQ Gradient A10 system (Millipore, Schwalbach, Germany). GSE was kindly provided by Kaden Biochemicals (Hamburg, Germany). (+)-CT was purchased from Applichem (Darmstadt, Germany). (–)-EC and β-glucuronidase from *Helix pomatia* were obtained from Sigma-Aldrich. L-ascorbic acid and NaEDTA were ordered from Carl Roth. Sephadex LH20 was purchased from GE Healthcare (Freiburg, Germany).

Procyanidin B1 (apple), B2, B5 (cocoa), B3 (barley), B4, B7 (GSE), C1 (cocoa), and A2 as well as Cinnamtannin B1 (both Litchi pericarp) were isolated from plant materials (given in brackets). A detailed description of isolation is given in [1]. The identity of standard substances was verified by mass spectrometric data and nuclear resonance spectroscopic data that are in accordance with literature data [33, 34, 35, 36, 37, 38, 39]. Stereochemistry was investigated by circular dichroism spectroscopy. Procyanidin B6, B8, and C2 were synthesized according to Delcour *et al.* and purified by column chromatography [1983]. The synthesis of 3′- and 4′-*O*-methyl derivatives of (+)-CT (3′OMCT and 4′OMCT) and (−)-EC (3′OMEC and 4′OMEC) was performed as described in literature [41]. After purification by preparative RP-HPLC, (where RP is reversed phase) identity was verified by MS and one dimensinal and two dimensional nuclear magnetic resonance spectroscopy [41, 42].

For the analysis of degradation products of procyanidins in urine, 3-phenylpropionic acid, 3-hydroxybenzoic acid, 4-hydroxybenzoic acid, 3-hydroxyphenylacetic acid, phloroglucinol, and hippuric acid were obtained from Merck (Darmstadt, Germany). 4-hydroxybenzoic acid methylester, sinapic acid, vanillic acid, and 3-L-phenyllactic acid were provided from Fluka (Buchs, Switzerland). 3-Hydroxyphenylpropionic acid was ordered from ABCR (Karlsruhe, Germany). Ferulic acid, protocatechuic acid, p-coumaric acid, m-coumaric acid, syringic acid, and N*,* O-bis(trimethylsilyl)acetamide (BSA) were obtained from Carl Roth. 3, 4-dihydroxyphenylacetic acid, 4-hydroxyphenylacetic acid, 4-hydroxymandelic acid monohydrate, 3, 4-dihydroxycinnamic acid, 4-hydroxyphenylpropionic acid, 3, 4-dihydroxyphenylacetic acid, 3, 4-dihydroxyhydrocinnamic acid, and homovanillic acid were provided from Sigma-Aldrich.

### 2.2 Animals, study design, and diet

Kinetic study with pigs was performed at the Institute of Animal Nutrition, Friedrich-Loeffler-Institute (FLI), Braunschweig, Germany. Treatments and experiments were conducted according to the European Community regulations concerning the protection of experimental animals and the guidelines of the Regional Council of Braunschweig, Lower Saxony, Germany (FileNumber 33.14-42502-04-037/08). Five castrated male pigs, crossbreed German Landrace × Pietrain, were kept individually in balance cages with a slitted floor connected to an appropriate-sized funnel and urine collection bottle. Body weight was approximately 46.6 ± 2.6 kg for pigs given mredGSE (*n* = 3) and 40.1 ± 3.0 kg for pigs of control group (*n* = 2). The daily feed amount of 1.5 kg was administrated in two equal portions at 7 a.m. and 2 p.m. Main diet constituents were wheat, barley, and soybean meal (for details see Supporting Information Table S1). At time point 0 h, mredGSE in a concentration of 250 mg/kg body weight was added to the feed of three pigs. The other two pigs were used as control group. Urine was collected 0, 3, 6, 9, 12, 24, 27, and 30 h after starting the kinetic study and stored at −80°C until analysis. Creatinine in urine samples was determined by the central laboratory of the university hospital of Muenster.

For preparation of the mredGSE, a Kromaton-200 Fast Centrifugal Partition Chromatograph (Kromaton Technologies, Sainte Gemmes sur Loire, France) equipped with a rotor volume of 200 mL was used. The separation was carried out at room temperature, at a speed of 1000 rpm and at a flow rate of 5 mL/min. A Wellchrom K-501 pump (Knauer, Berlin, Germany) was used to pump liquids. The used solvent system consisted of tert-butylmethylether/ethyl acetate/water (4/6/10, v/v/v). The lower phase was used as mobile phase. Elution was monitored with a Wellchrom K-2501 UV-VIS-detector (Knauer) at 280 nm. Fractions were collected with an Isco Foxy fraction collector (Teledyne, Lincoln, NE).

Two grams of GSE were dissolved in a 1:1 mixture (8 mL) of upper and lower phase and injected into the rotating centrifugal partition chromatograph. After elution of procyanidins with a degree of polymerization ≥ 2 flavan-3-ols elute. The separation between dimeric procyanidins and flavan-3-ols was checked by thin-layer chromatography on silica with ethyl acetate/formic acid/water (90/5/5, v/v/v) as mobile phase. Detection was performed by vanillin/hydrochloric acid spray reagent. The fractions containing dimers up to polymers were combined and freeze dried after removal of the organic solvent.

### 2.3 Analysis of feed and mredGSE

Removal of flavan-3-ols from GSE was investigated by HPLC-FLD (where FLD is fluorescence detector). The system consisted of a DGU-20A3 degasser, two LC-10AT pumps, a SIL-10AF autosampler, a CTO-10ASVP column oven and a RF-10A XL fluorescence detector (all from Shimadzu, Duisburg, Germany). Separation was performed according to Kelm *et al.* with slight modifications [2006]. A LiChrospher 100 Diol column, 250 × 4.6 mm with 5-μm particle size (Merck) was used as stationary phase. The mobile phase consisted of a linear gradient of acetonitrile/formic acid (99/1, v/v) and methanol/water/formic acid (95/4/1, v/v/v) (B) with a rise over 35 min from 0 to 40% B that was held isocratically for 10 min. Flow rate was 0.8 mL/min at a column temperature of 35°C and injection volume was 5 μL (dissolved in acetonitrile/methanol 3/1, v/v). Excitation wavelength of the fluorescence detector was 276 nm, the emissions were monitored at 316 nm.

Procyanidins in mredGSE and feed were analyzed with LC-MS/MS (where LC is liquid chromatography) and echo-peak technique. The following standard substances were used: procyanidin B1, B2, B3, B4, B5, B6, B7, B8, C1, C2, and A2 as well as Cinnamtannin B1. Detailed description is given in [1]. Phenolic acids in the mredGSE and feed were analyzed on a HP6890 gas chromatograph coupled to a HP5973 mass spectrometer (both Agilent, Waldbronn, Germany) and a Combi PAL autosampler (CTC Analytics, Zwingen, Switzerland). Chromatographic separation was performed on 30 m × 0.25 mm id RTX-5 column with a thickness of 0.1 μm (Restec, Bad Homburg, Germany) using 1 mL/min helium as carrier gas. The injector temperature was set at 280°C, and the injection volume was 1 μL with split injection (1:20). The temperature gradient published by Grün *et al.* was used for the separation [2008]. The transfer line was heated at 320°C. The mass spectrometer was operated in the electron impact mode (EI, 70 eV). Source temperature was set at 230°C and the quadrupole was heated at 150°C. Mass spectra were acquired in the full scan mode ranging from *m/z* 55 to 600 with a scan rate of 2.7 scan/s. Identity of compounds was verified by using the NIST mass spectral library (where NIST is National Institute of Standards and Technology) and search program. The following ions were used for quantification if necessary: 4-hydroxybenzoic acid methylester as internal standard (IS) (*m/z* 209), 3-phenylpropionic acid (*m/z* 207), 3-hydroxybenzoic acid (*m/z* 267), 3-phenyllactic acid (*m/z* 193), 3-hydroxyphenylacetic acid (*m/z* 281), 4-hydroxybenzoic acid (*m/z* 267), 4-hydroxyphenylacetic acid (*m/z* 281), phloroglucinol (*m/z* 342), 3-hydroxyphenylpropionic acid (*m/z* 205), 4-hydroxyphenylpropionic acid (*m/z* 310), vanillic acid (*m/z* 297), homovanillic acid (*m/z* 326), 4-hydroxymandelic acid (*m/z* 267), protocatechuic acid (*m/z* 370), 3, 4-dihydroxyphenylacetic acid (*m/z* 384), m-coumaric acid (*m/z* 293), syringic acid (*m/z* 327), p-coumaric acid (*m/z* 293), 3, 4-dihydroxycinnamic acid (*m/z* 398), ferulic acid (*m/z* 338), 3, 4-dihydroxycinnamic acid (*m/z* 396), and sinapic acid (*m/z* 368). Identity of substances was confirmed by two further ions for each substance.

For qualitative analysis of free, conjugated, and bound phenolic acids in feed, the method published by Li *et al.* was modified [2008]. The residues of the different ethylacetate extracts were derivatized with 150 μL pyridine and 150 μL BSA for 25 min at 55°C before GC-MS (where GC is gas chromatography) analysis.

### 2.4 Analysis of urine samples

For HPLC-ESI-MS/MS (where ESI is electrospray ionization) analysis of flavan-3-ols and procyanidins in urine samples, an Agilent 1100 series HPLC (Agilent) was linked to an API 4000 QTrap mass spectrometer (Applied Biosystems, Darmstadt, Germany). Chromatographic separation was performed on a 250 × 2 mm, 5-μm, LiChrospher RP 18 column (Merck) using a binary gradient of water/formic acid (99.9/0.1, v/v) and acetonitrile/formic acid (99.9/0.1, v/v) at a flow rate of 300 μL/min. The following gradient at 25°C was used: 11% B (0 min), 11% B (5 min), 16% B (15 min), 41% B (30 min). Thirty microlitres of sample were injected. Injector temperature was set at 7°C. The mass spectrometer was operated in the multiple reaction monitoring (MRM) mode detecting negative ions. For fragmentation of the molecular ions into the specific fragment ions, nitrogen was used as collision gas. Zero-grade air served as the nebulizer gas (30 psi) and was heated at 350°C as turbo gas for solvent drying (45 psi). Ion spray voltage was set at −4500 V. The following transition reactions were monitored for a duration of 150 ms each (declustering potential [DP], collision energy [CE], and collision exit potential [CXP] given in brackets): flavan-3-ols, 288.9–108.9 (DP, −75 V; CE, −39 V; CXP, −5 V); monomethyl derivates of flavan-3-ols, 302.9–243.7 (DP, −85 V; CE, −24 V; CXP, −7 V); dimeric compounds, 577.1–407.0 (DP, −85 V; CE, −34 V; CXP, −11 V), and trimeric compounds 865.1–407.1 (DP, −110 V; CE, −58 V; CXP, −21 V). For quantification, standard solutions containing CT, EC, and their 3′- and 4′-*O*-methyl derivatives (5–400 ng/mL, respectively 250–5000 ng/mL for samples after hydrolysis), procyanidin B1, B2, B3, B4, C1 (5–400 ng/mL, both cases), and IS procyanidin B6 were prepared and analyzed by HPLC-ESI-MS/MS. For calculation of calibration curves, the peak area ratios of the analytes to the IS were plotted against the concentration of analytes. For kinetic curves, the concentrations of analytes were referred to the creatinine concentrations of the corresponding samples. For the determination of the total excretion, concentrations at the different time points were multiplied with the corresponding urine volumes and added. Density of urine was assumed to be 1 in all cases.

For analysis of flavan-3-ols, their methyl derivatives, and procyanidins in urine, a column (6 × 1 cm) was filled with Sephadex LH20 up to a filling height of 4 cm. The column was conditioned with 16 mL water/methanol (95/5, v/v). To 800 μL urine, 80 μL ascorbic acid/NaEDTA solution (20 g/100 mg in 100 mL water) and 80 μL IS solution (Procyanidin B6, 250 ng/mL in water) were added. After loading of the sample, the column was washed with 7 mL water/methanol (95/5, v/v). Analytes were eluted with 15 mL acetone/water (70/30, v/v). After evaporation to dryness, the residue was dissolved in 200 μL 0.1% formic acid/acetonitrile (90/10, v/v). Quantification was performed by HPLC-MS/MS. Recovery rates were calculated by adding all standard substances in a concentration of 10, 50, and 100 ng/mL to pig urine of the control group. Results were corrected by natural occurring concentrations.

To determine the proportion of conjugated compounds, samples were treated with β-glucuronidase and analyzed for a second time. Therefore 60 μL sodium acetate solution (1.5 M, pH 4.8) and 30 μL β-glucuronidase solution (1500 U, in 150 mM sodium acetate solution, pH 4) were added to 800 μL urine and incubated at 37°C for 45 min. The further sample clean up was performed according to the protocol described above. For the analysis of conjugated compounds of CT, EC, 3′OMCT, 4′OMCT, 3′OMEC, and 4′OMEC, an IS solution with a higher concentration (Procyanidin B6, 5000 ng/mL in water) was used. Injection volume was reduced to 10 μL.

For qualitative identification of original conjugated compounds, a Thermo Orbitrap XL mass spectrometer with an Accela pump and injector (all Thermo Fisher Scientific, Bremen, Germany) was used. The chromatographic separation was performed by using the same stationary phase and eluents as described for the API 4000Q-Trap system. A linear gradient from 5% B to 25% for 30 min at 25°C at a flow rate of 300 μL/min was used. Injection volume was 50 μL. Heated negative electrospray ionization mode (HESI^−^) was used. Further adjustments were as follows: capillary voltage −37 V, ionization voltage 3.3 kV, capillary temperature 225°C, sheat gas flow 40, aux gas flow 20, sweep gas flow 10, tube lens −179 V. Resolution was 30 000. For structural elucidation, experiments in collision-induced dissociation mode (CID) with a normalized CE of 13% were performed. Urine samples were centrifuged (10 min, 13 000 × *g*, 20°C) and directly used for HPLC-FTMS analysis.

The analysis of hippuric acid in urine was performed on the same HPLC system as described in section 2.3 for the analysis of the mredGSE. Instead of the fluorescence detector, a SPD-1120A DAD (Shimadzu) was used. Hippuric acid was analyzed isocratically with 0.5% formic acid/methanol (90/10, v/v) as mobile phase on a Lichrospher 60 RP-select B column, 125 × 4 mm with 5-μm particle size (Merck). Column temperature was set at 25°C, an injection volume of 10 μL, and a flow rate of 1 mL/min were used. Identity was checked by comparison of UV spectra to that one of the reference. Quantification was performed at 232 nm by using a calibration curve for hippuric acid.

The sample preparation was performed according to Gonthier *et al*. with some modifications [2003]. In brief, 20 μL sodium acetate solution (1.5 M, pH 4.8) and 10 μL ß-glucuronidase solution (500 U, in 150 mM sodium acetate, pH 4.8) were added to 170 μL urine and incubated for 90 min at 37°C. After addition of 500 μL MeOH/HCl (200 nmol) and 300 μL water samples were centrifuged (10 min, 13 000 × *g*, 20°C). The supernatant was used for the HPLC-DAD analysis. Recovery rates were calculated after addition of hippuric acid in concentrations of 1–2.5 mg/mL to pig urine. Results are between 103% and 105% (after subtraction of naturally occurring concentrations).

For the analysis of phenolic acids in urine, the same GC-MS method as described in section 2.3 was used. Quantification was performed by using calibration curves of standard substances ranging from 5–50 μg/mL and 4-hydroxybenzoic acid methylester as IS.

A clean up procedure published by Grün *et al.* was modified [2008]. Hydrolysis of conjugated compounds was performed by adding 60 μL sodium acetate solution (1.5 M, pH 4.8) and 30 μL β-glucuronidase solution (1500 U, in 150 mM sodium acetate solution, pH 4.8) to 500 μL urine. After an incubation at 37°C for 90 min, reaction was stopped with 20 μL 6 M hydrochloric acid and 50 μL IS solution (200 μg/mL in methanol/water 50/50, v/v) was added. Phenolic acids were extracted by shaking with 2 mL ethylacetate for 1 min. After centrifugation (10 min, 1400 × *g*, 4°C), the organic phase was separated. The extraction was repeated two times and the collected organic phases were combined. After evaporation to dryness, the residue of organic phases was silylated by addition of 150 μL pyridine and 150 μL BSA for 25 min at 55°C. The derivatized samples were measured by GC-MS. For calculation of recovery rates, the phenolic acids, which were identified in the urine qualitatively, were added to pig urine in a concentration of 5, 10, 25 μg/mL. Recovery rates vary between 80% and 105% after subtraction of naturally occurring concentration with exception of homovanillic acid and 4-hydroxymandelic acid. They show slightly higher, respectively, lower recovery rates (for details see Supporting Information Fig. S2).

### 2.5 Statistics

All presented data are given as mean ± SD. Differences were determined using the unpaired Student's *t*-test with p ≤ 0.05 as significant different results (with Excel 2003).

## 3 Results

### 3.1 Analysis of feed and mredGSE

**Figure 2:**
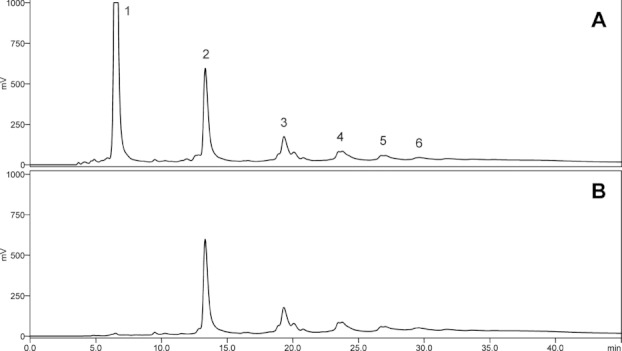
NP-HPLC-FLDchromatograms (ex: 276 nm, em: 316 nm) of GSE before (A) and after reduction of monomeric compounds (flavan-3-ols) (B) by using centrifugal partition chromatography: monomeric (1), dimeric (2), trimeric (3), tetrameric (4), pentameric (5), and hexameric (6) compounds.

The NP-HPLC-FLD chromatogram of the mredGSE shows the successful reduction of the peaks corresponding to the flavan-3-ols CT and EC in comparison to the unmodified GSE (see Fig. 2). Removal of the flavan-3-ols by CPC was also confirmed by HPLC-ESI-MS/MS. Signals corresponding to CT and EC were under the limit of quantification with a signal-to-noise ratio of 4.2 and 3.7, respectively. No gallylated flavan-3-ols could be identified by HPLC-MS/MS. Procyanidin B1 (1528.9 ± 31.7 mg/100 g), B2 (1706.5 ± 38.0 mg/100 g), B3 (752.9 ± 53.0 mg/100 g), B4 (723.4 ± 48.2 mg/100 g), and C1 (919.1 ± 25.3 mg/100 g) were identified as main procyanidin constituents in mredGSE. No phenolic acids were detectable by GC-MS.

The analysis of procyanidins in the basic feed administrated to pigs led to the following results: CT (2.5 ± 0.1 mg/100 g), procyanidin B3 (3.9 ± 0.3 mg/100 g), B6 (0.2 ± 0.0 mg/100 g), and C2 (1.2 ± 0.1 mg/100 g). The small amounts of procyanidins in the feed samples are coming from the barley in the diet. Additionally, 4-hydroxybenzoic acid, 4-hydroxyphenylacetic acid, vanillic acid, syringic acid, p-coumaric acid, ferulic acid, and sinapic acid were identified in the feed by GC-MS. All phenolic acids were present in free, conjugated, and bound form.

### 3.2 Analysis of urine samples

The recovery rates for solid phase extraction with Sephadex LH20 were determined by adding standard substances (CT, EC, 3′OMCT, 4′OMCT, 3′OMEC, 4′OMEC, procyanidin B1, B2, B3, B4, and C1) to pig urine of the control group in concentrations of 10, 25, and 100 ng/mL. The resulting recovery rates are ranging from 70 to 115% for CT, EC, and procyanidins. For the methyl derivatives of CT and EC, recovery rates are about 50% or lower (for details see Supporting Information Fig. S3).

**Figure 3:**
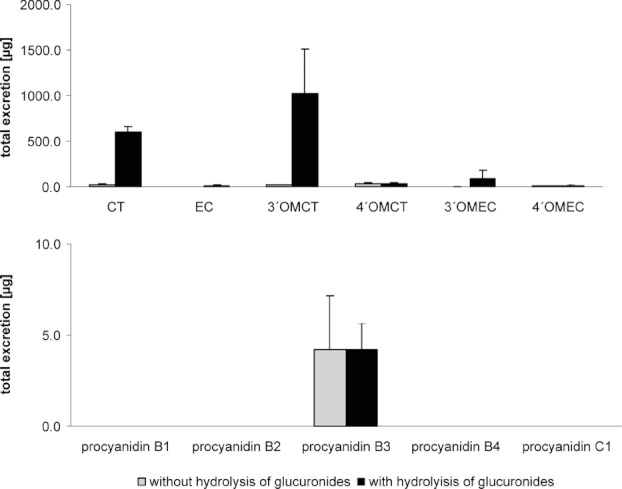
Total excretion of flavan-3-ols, methylated flavan-3-ols, and procyanidins from pigs of the control group (*n* = 2) 0–30 h after starting the kinetic study with and without hydrolysis of glucuronides (means ± SD).

In urine samples of the control group (*n* = 2), CT, EC, procyanidin B3, 3′OMCT, 4′OMCT, 3′OMEC, and 4′OMEC were detectable (see Fig. 3). After treatment with β-glucuronidase, distinct higher values for flavan-3-ols and their methyl derivatives could be observed. Total excretion of EC and 3′OMEC is much lower compared to CT and 3′OMCT, respectively. Figure 3 presents no significant change in the total excretion of procyanidin B3 after treatment with β-glucuronidase. Procyanidin B3 (about 4 μg) was excreted to a much lower extent in comparison to CT.

**Figure 4:**
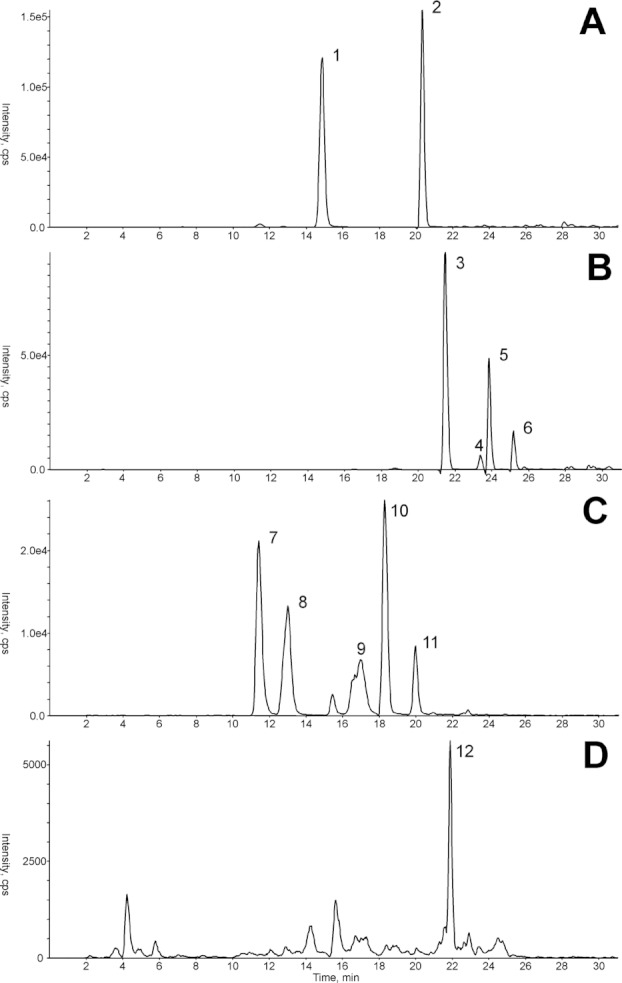
RP-HPLC-ESI-MS/MS chromatogram of a urine sample of a pig given mredGSE 3 h after starting the kinetic study. Sample was treated with β-glucuronidase. (A) Shows flavan-3-ols with MRM *m/z* 288.9–108.9 [M-H]^−^, (B) methyl derivatives of flavan-3-ols with MRM *m/z* 302.9-243.7 [M-H]^−^, (C) dimeric procyanidins with MRM *m/z* 577.1-407.0 [M-H]^−^, (D) trimeric procyanidins with MRM *m/z* 865.1-407.1 [M- H]^−^. CT (1), EC (2), 3′OMCT (3), 4′OMCT (4), 3′OMEC (5), 4′OMEC (6), procyanidin B1 (7), B3 (8), B4 (9), B2 (10), B6 (IS) (11), and C1 (12).

**Figure 5:**
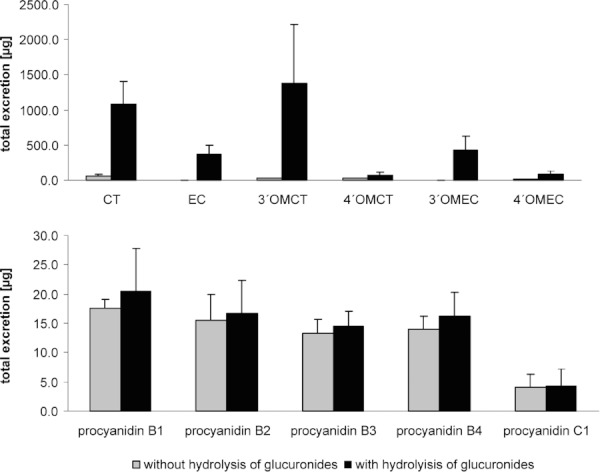
Total excretion of flavan-3-ols, methylated flavan-3-ols, and procyanidins from pigs given mredGSE (*n* = 3) 0–30 h after starting the kinetic study with and without hydrolysis of glucuronides (means ± SD).

In urine samples from pigs (*n* = 3) that were feed with mredGSE (250-mg/kg body weight) CT, EC, their methyl derivatives, procyanidin B1, B2, B3, B4, and C1 were identified. Figure 4 shows a HPLC-ESI-MS/MS chromatogram of a urine sample (treated with β-glucuronidase) of a pig 3 h after feeding mredGSE. Total excretion of procyanidins ranged between 13 and 20 μg for dimeric compounds, and about 5 μg for the trimeric procyanidin C1 (see Fig. 5). After treatment with β-glucuronidase, no significant increase for total excretion of procyanidins was detectable. Total excretion of CT, EC, and their methylated derivatives was also distinctly higher after hydrolysis of the glucuronides. 3′OMCT and 3′OMEC were excreted to a higher extent in comparison to the 4′-*O*-methyl derivatives (see Fig. 5). Total excretion of flavan-3-ols (CT 1000 μg, EC 370 μg) and their methyl derivatives is also higher compared to the total excretion of procyanidins (about 5–20 μg).

A comparison between control group and pigs given mredGSE showed a higher excretion of CT, EC, and their methyl derivatives for the later group in all cases. The difference between pigs given mredGSE and control group is about 400 μg for CT and for EC. A similar difference was observed for 3′OMCT and 3′OMEC.

**Figure 6:**
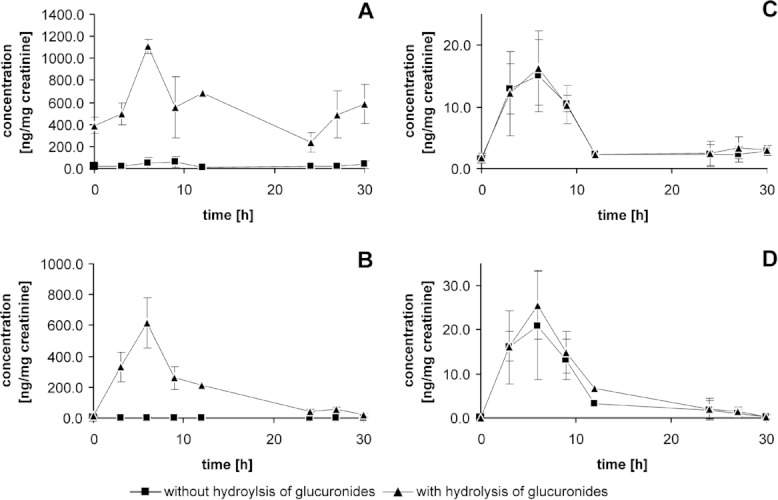
Kinetic curves of CT (A), EC (B), procyanidin B3 (C), and B4 (D) in urine of pigs given mredGSE (*n* = 3) with and without hydrolysis of glucuronides. Data expressed as means ± SD.

Figure 6 shows the concentrations (corrected by creatinine) of CT, EC, procyanidin B3, and B4 with and without hydrolysis of glucuronides for pigs given mredGSE as a function of time. Maximum concentration of CT (Fig. 6A) and EC (Fig. 6B) was reached 6 h after administration of mredGSE. CT shows a second, but weaker maximum, after 30 h. Maximum concentration of procyanidin B3 (Fig. 6C) and B4 (Fig. 6D) was at the same time point as for CT and EC (6 h after administration of mredGSE). In the case of procyanidin B3, very low concentrations were detectable at the starting point of kinetic study and also 30 h after administration of mredGSE because of the presence of procyanidin B3 in the feed in contrast to procyanidin B4. Kinetic curves for 3′OMCT and 4′OMCT show a similar course as obtained for CT. Concentration–time functions of 3′OMEC and 4′OMEC have a high similarity to that one of EC. Concentrations of procyanidin B1, B2, and C1 reached their maximum after 6 h and dropped to almost zero such as in the case of procyanidin B4 (for kinetic curves of further analytes see Supporting Information Figs. S4–S10,). About 0.01–0.02% of the administrated dimeric procyanidins were excreted with urine. In the case of the trimeric procyanidin C1, the ratio of excretion to administration is 0.004%. Data are calculated for a pig with 46.6 kg body weight that corresponds to 11.6 g mredGSE. Three feedings each about 0.75 kg are the basis for calculation (see Table 1). In comparison, 1% or respectively 3% CT (only CT or respectively CT and methyl derivates, both after hydrolysis) was excreted from pigs of the control group as a consequence of CT in the feed.

**Table 1:** Total excretion of procyanidins by pigs given mredGSE (*n* = 3) as percentage (%) of the sum of the administrated procyanidins from the supplement (mredGSE) and from the feed (30-h urine collection time, mean 46.6 kg body weight, corresponding 11.6 g mrdGSE) (means ± SD)

	Procyanidins administrated by		Procyanidins sum (mg)	Total excretion (μg)	Total excretion (%)
	mredGSE (mg)	Feed (mg)			
Procyanidin B1	177.4 ± 3.7		177.4 ± 3.7	20.5 ± 7.3	0.012 ± 0.004
Procyanidin B2	198.0 ± 4.4		198.0 ± 4.4	16.7 ± 5.7	0.008 ± 0.003
Procyanidin B3	87.3 ± 6.1	87.8 ± 6.8	175.1 ± 12.9	14.4 ± 2.6	0.008 ± 0.001
Procyanidin B4	83.9 ± 5.6		83.9 ± 5.6	16.1 ± 4.1	0.019 ± 0.005
Procyanidin C1	106.6 ± 2.9		106.6 ± 2.9	4.3 ± 2.8	0.004 ± 0.003

**Figure 7:**
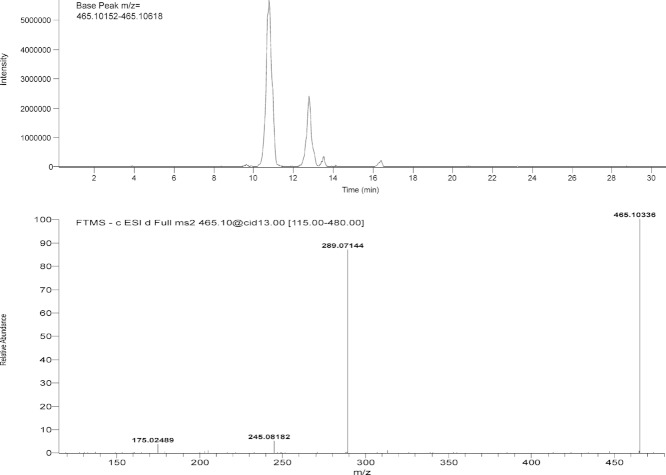
RP-HPLC-FTMS chromatogram of a urine sample of a pig given mredGSE 3 h after starting of the kinetic study. Peaks with *m/z* 465.1034 [M-H]^−^ correspond to glucuronides of flavan-3-ols with a calculated mass of *m/z* 465.1039 [M-H]^−^ (above). MS/MS spectrum of the first peak with m/z 465.10 [M-H]^−^ with normalized collision energy (CID) 13% (below). Signal with m/z 289.0714 [M-H]^−^ corresponds to flavan-3-ol (calculated mass m/z 289.0718 [M-H]^−^). The second peak shows the same fragmentation pattern.

Glucuronides of flavan-3-ols and their methyl derivatives were confirmed by the direct measurement of urine samples using HPLC-FTMS. Figure 7 presents the HPLC-FTMS chromatogram of an urine sample of one pig 3 h after administration of mredGSE. Two peaks with *m/z* 465.1034 [M-H]^−^ corresponding to flavan-3-ol glucuronides with calculated *m/z* 465.1039 [M-H]^−^ were detectable by their exact mass. MS/MS fragmentation pattern shows a fragment of *m/z* 289.0714 [M-H]^−^ corresponding to the flavan-3-ol aglycon. Based on the mass spectrometric data, the two peaks are tentatively identified as CT- and EC-glucuronide. HPLC-FTMS chromatograms of the methyl derivatives of CT and EC gave similar results (for details see Supporting Information Fig. S11). No methyl derivatives and glucuronide adducts of procyanidins could be identified.

**Figure 8:**
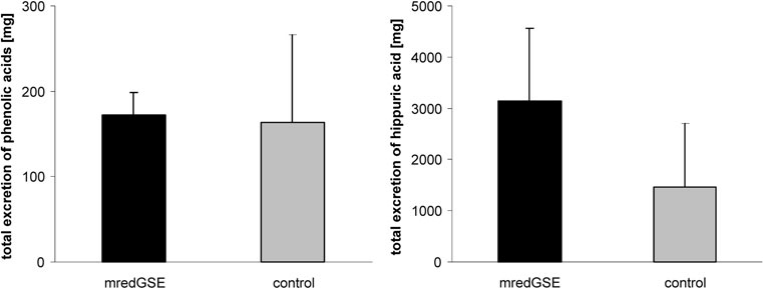
Total excretion of phenolic acids as sum (left) and hippuric acid (right) in comparison between pigs given mredGSE (*n* = 3) and pigs of the control group (*n* = 2) 0–30 h after starting the kinetic study (means ± SD).

As possible metabolites of procyanidins, phenolic acids and hippuric acid were analyzed by GC-MS or HPLC-DAD. The following phenolic acids were identified and quantified: 3- and 4-hydroxybenzoic acid, 3-phenyllactic acid, 3- and 4-hydroxyphenylacetic acid, 3-hydroxyphenylpropionic acid, vanillic acid, homovanillic acid, 4-hydroxymandelic acid, protocatechuic acid, 3, 4-dihydroxyphenylacetic acid, ferulic acid, 3, 4-dihydroxycinnamic acid, and sinapic acid. Figure 8 presents the results for quantification of phenolic acids as sum of all detectable excreted phenolic acids. No trend can be seen between pigs given mredGSE and the control group. Kinetic curves of the single phenolic acids present also no trend (data not shown). Total excretion of hippuric acid tend to be higher for pigs given mredGSE in comparison to the control group.

## 4 Discussion

The investigation of urinary excretion and metabolism of procyanidins in the absence of flavan-3-ols was the main focus of this study. For this purpose, it was necessary to develop a method for the reduction of flavan-3-ols in a commercial GSE based on CPC. A system of tert-butylmethylether/ethyl acetate/water allowed an efficient separation of procyanidins and flavan-3-ols and resulted in the large-scale production of mredGSE (1.7 g/CC run) that was used for the feeding study.

This mredGSE was administrated to pigs, which have a gastric tract similar to human ones, at a dose of 250 mg/kg body weight. Oral dosing of procyanidins via feed of pigs was chosen as most realistic application form instead of intragastric injections that were used in some other studies.

The newly developed HPLC-ESI-MS/MS method for analysis of procyanidins in urine based on a solid phase extraction with Sephadex LH20 enabled quantification of even low concentrations of flavan-3-ols and procyanidins (10 ng/mL) with acceptable recovery rates above 70%. Sephadex LH20 as stationary phase offered the great advantage of a higher selectivity for procyanidins in comparison to often-used RP material. Thereby, matrix effects during HPLC-MS/MS measurement can be reduced effectively. Dimeric procyanidin B6 is a suitable IS as it is not detectable in pig urine and can be easily obtained by a single-step chemical synthesis. The concentrations of procyanidin B6 in feed seems to be too low for an identification in urine samples. A possible explanation for the observed lower recovery rates of methyl derivatives of CT and EC is the lower polarity due to the methylation of the hydroxyl groups.

By using this methodology, procyanidin B1, B2, B3, B4, and C1 were identified in urine of pigs that were fed with the mredGSE. The identification of dimeric procyanidins is in good accordance with literature. Baba *et al.* were also able to identify procyanidin B2 in urine after administration of procyanidin B2 to rats [2002]. In further studies, dimeric procyanidins were also identified in urine of rats after ingestion of GSE [11, 12], cocoa [16], and apple-condensed tannins [10]. Also, in human urine, procyanidin B2 was detectable after cocoa intake [16]. Identification and especially quantification of procyanidin C1 in urine was possible due to the high sensitivity of the used analytical procedure. Prasian *et al.* and Tsang *et al.* were also able to identify trimeric procyanidins in rat urine after oral administration of GSE [11, 12]. Taking these results together with those of Shoji *et al*. [2006], we could clearly show that trimeric compounds can be absorbed. Quantification of procyanidins in urine led to the following conclusion. The total excretion varies between 14 and 20 μg for dimeric procyanidins and was about 5 μg for procyanidin C1. Therefore, absorption of dimeric and trimeric procyanidins is probably poor considering the total excretion as percentage of total intake (supplemented via mredGSE plus procyanidins from feed) ranging from 0.004% for trimeric procyanidins to 0.019% for dimeric procyanidins. Similar low ratios (in average 0.5%) were observed by Tsang *et al.* after administration of a higher dose of GSE (1-g/kg body weight) to rats [2005]. The extent of absorption seems to be depending on the degree of polymerization. This trend was also observed by Shoji *et al.* after administration of apple condensed tannins where dimeric procyanidins showed higher plasma concentration than trimers [2006]. An interesting observation was made in the case of the control group. Procyanidins can be even absorbed from normal feed. One constituent of pig feed is barley that contains procyanidin B3. In urine of pigs from the control group procyanidin B3 could be quantified. This observation is of interest for the transfer of the result to human diet. Even the normal concentrations of procyanidins in foods are sufficient enough for an absorption. Results are in contrast to other studies detecting no procyanidins in blood and urine [17, 18]. After administration of sorghum and extrudated sorghum also to pigs, no dimeric procyanidins were detectable in urine and plasma [46]. Administrated concentrations of procyanidins are low and therefore levels of absorbed procyanidins might be too low for a detection by the used methodology.

In comparison to procyanidins, flavan-3-ols, CT, and EC were excreted in distinct higher concentrations that indicate their higher bioavailability. Ratio of total excretion to administrated amounts for CT is about 1%, respectively, 3% (only CT, respectively, CT and methyl derivatives, both after hydrolysis) for pigs of control group. These results underline the different absorption rates between flavan-3-ols and procyanidins in connection with the degree of polymerization as observed in former studies [10, 11]. This was also confirmed by experiments with an *in situ* perfusion model of the small intestine of rats. Absorption rate of dimeric procyanidins (A- and B-type) was 5–10% of that of EC [47].

Kinetic curves of CT for pigs given mredGSE showed increasing concentrations after every feeding (after 6–12 h and after 30 h). Interestingly, the increase of CT concentration is higher after additional administration of mredGSE to feed. This trend was also obvious in a comparison of total excretion of flavan-3-ols and their methyl derivatives between pigs given mredGSE and pigs of the control group. The increased excretion can result from a degradation of high-molecular-weight procyanidins under release of smaller units. A possible degradation of higher oligomeric and polymeric procyanidins in the stomach can be one explanation. In incubation experiment with simulated gastric juice, Spencer *et al.* observed a degradation of higher oligomeric procyanidins (trimers to hexamers) to flavan-3-ols and dimeric procyanidins [2000]. Both are probably stable under the conditions in the stomach and can be seen as final products of a possible degradation of procyanidins by gastric juice. Besides a possible degradation of procyanidins in the stomach, these substances can also be degradated in other parts of the gastrointestinal tract, such as the small intestine [2001]. Also *in vivo* studies underline the fact of a possible degradation of procyanidins during the transit of the gastrointestinal tract. After the administration of procyanidin B2 to rats, Baba *et al.* observed degradation to flavan-3-ols by the identification of EC and their methyl derivatives in plasma [2002]. Kahle *et al.* investigated a possible degradation of procyanidins by administration of apple juice to ileostomy subjects. A reduction of the mean degree of polymerization of procyanidins in the ileostomy effluent in comparison to the apple juice was observed [2007].

Flavan-3-ols were metabolized to a greater extent by methylation and glucuronidation in comparison to procyanidins. About half of the total CT and EC was detectable in form of the methyl derivatives. Independent of methylation, more than 90% of the analytes were conjugated with glucuronic acid. Kuhnle *et al.* demonstrated methylation and glucuronidation of CT and EC in an isolated rat small intestine perfusion model [2000]. After incubation of epicatechin-3-gallate with rat hepatic and intestinal microsomes, a glucuronidation of the A and B ring of the EC moiety was observed [51]. Also *in vivo* studies confirmed the extensive metabolism of CT and EC, as in the case of Baba *et al.* after administration of CT, EC, and their mixture to rats [2001]. In contrast to flavan-3-ols, concentrations of procyanidins were nearly the same after treatment with β-glucuronidase. This was confirmed by HPLC-FTMS and in addition no methyl derivatives could be identified. These result are in accordance with literature. In an *in situ* perfusion model of the small intestine of rats, Appeldorn *et al.* observed an absorption of B- and A-type procyanidins without conjugation or methylation [47].

Concerning possible low molecular weight metabolites, a trend of an increased excretion of hippuric acid was observed. A similar effect is described after feeding of wine polyphenols to rats and for humans after intake of cocoa powder [16, 30]. In contrast to hippuric acid, no trend was obvious for phenolic compounds. Phenolic acids in sum or as single compound did not increased significantly. Some characteristic metabolites such as phloroglucinol found in our previous study [27] were not detectable in the urine samples. The reason of this might be the low dose of mredGSE (250 mg/kg body weight) compared with other studies. As a result, changes in the concentrations of phenolic acids are too low in comparison to the naturally occurring levels as a result of the degradation of phenolic constituents of the feed. In addition, interindividual differences have to be considered. It could be shown in the pig cecum model that the intestinal degradation of procyanidin B2 is highly depending on the individual intestinal microbiota. Furthermore, the microbiota was not able to degradate procyanidin C1 [27]. These results may be also an explanation for the missing increase of phenolic acids especially in the view of the composition of the mredGSE. The mredGSE contains mainly high oligomeric and polymeric procyanidins and nearly no flavan-3-ols that could be easily degradated to phenolic acids.

In conclusion, after intake of a mredGSE by pigs, flavan-3-ols, their methyl derivatives, dimeric and trimeric procyanidins were detectable in urine. This fact suggests an absorption of procyanidins by pigs, which should be confirmed in further studies by analysis of blood samples. Excretion was depending on degree of polymerization and very poor for dimeric and trimeric procyanidins. Due to the similarity of the gastrointestinal tract of pigs compared to humans, an absorption of procyanidins from the diet by humans seems possible. Especially in the focus of the observation that the pigs of the control group excreted procyanidin B3 resulting from the barley in the feed. A degradation of procyanidins with high degree of polymerization under release of flavan-3-ols is possible. Flavan-3-ols were metabolized by glucuronidation and methylation, procyanidins not. In the case of low molecular weight metabolites, only a trend of an increase of hippuric acid can be observed.

## Abbreviations

3′OMCT: 3′-*O*-methylcatechin
4′OMCT: 4′-*O*-methylcatechin
3′OMEC: 3′-*O*-methylepicatechin
4′OMEC: 4′-*O*-methylepicatechin
BSA: *N, O*-bis(trimethylsilyl)acetamide
CPC: centrifugal partition chromatography
CT: catechin
EC: epicatechin
GSE: grape seed extract
mredGSE: monomer reduced grape seed extract
NaEDTA: sodium salt of ethylenediamine tetraacetic acid

## Notes

Disclaimer: Supplementary materials have been peer-reviewed but not copyedited.

Figure S1. Structures of methyl derivatives of catechin and epicatechin: 3′-O-methyl-(+)-catechin (1), 4′-O-methyl-(+)-catechin (2), 3′-O-methyl-(–)-epicatechin (3), and 4′-O-methyl-(–)-epicatechin (4). Figure S2. Recovery rates (%) of phenolic acids in pig urine after subtraction of naturally occurring concentrations. Figure S3. Recovery rates (%) of flavan-3-ols, methyl derivates of flavan-3-ols, and procyanidins in pig urine after subtraction of naturally occurring concentrations. Figure S4. Kinetic curve of procyanidin B1 in urine of pigs given mredGSE (*n*=3) with and without hydrolysis of glucuronides. Data expressed as means ± SD. Figure S5. Kinetic curve of procyanidin B2 in urine of pigs given mredGSE (*n*=3) with and without hydrolysis of glucuronides. Data expressed as means ± SD. Figure S6. Kinetic curve of procyanidin C1 in urine of pigs given mredGSE (*n*=3) with and without hydrolysis of glucuronides. Data expressed as means ± SD. Figure S7. Kinetic curve of 3′OMCT in urine of pigs given mredGSE (*n*=3) with and without hydrolysis of glucuronides. Data expressed as means ± SD. Figure S8. Kinetic curve of 4′OMCT in urine of pigs given mredGSE (*n*=3) with and without hydrolysis of glucuronides. Data expressed as means ± SD. Figure S9. Kinetic curve of 3′OMEC in urine of pigs given mredGSE (*n*=3) with and without hydrolysis of glucuronides. Data expressed as means ± SD. Figure S10. Kinetic curve of 4′OMEC in urine of pigs given mredGSE (*n*=3) with and without hydrolysis of glucuronides. Data expressed as means ± SD. Table S1. Composition of the diet (g/kg) that was fed to pigs (1.5 kg per day in two equal portions) of the control group (*n*=2) and the group given mredGSE (*n* = 3).
