# Supplementary material for: Urinary excretion and metabolism of procyanidins in pigs
Source: Mol Nutr Food Res. 2012 Apr 12;56(4):653–65. doi: 10.1002/mnfr.201100471 (PMC3494987; doi:10.1002/mnfr.201100471)
Supplement: Supplementary file 1 — Figure S1. Structures of methyl derivatives of catechin and epicatechin: 3′-O-methyl-(+)-catechin (1), 4′-O-methyl-(+)-catechin (2), 3′-O-methyl-(–)-epicatechin (3), and 4′-O-methyl-(–)-epicatechin (4). Figure S2. Recovery rates (%) of phenolic acids in pig urine after subtraction of naturally occurring concentrations. Figure S3. Recovery rates (%) of flavan-3-ols, methyl derivates of flavan-3-ols, and procyanidins in pig urine after subtraction of naturally occurring concentrations. Figure S4. Kinetic curve of procyanidin B1 in urine of pigs given mredGSE (n=3) with and without hydrolysis of glucuronides. Data expressed as means ± SD. Figure S5. Kinetic curve of procyanidin B2 in urine of pigs given mredGSE (n=3) with and without hydrolysis of glucuronides. Data expressed as means ± SD. Figure S6. Kinetic curve of procyanidin C1 in urine of pigs given mredGSE (n=3) with and without hydrolysis of glucuronides. Data expressed as means ± SD. Figure S7. Kinetic curve of 3′OMCT in urine of pigs given mredGSE (n=3) with and without hydrolysis of glucuronides. Data expressed as means ± SD. Figure S8. Kinetic curve of 4′OMCT in urine of pigs given mredGSE (n=3) with and without hydrolysis of glucuronides. Data expressed as means ± SD. Figure S9. Kinetic curve of 3′OMEC in urine of pigs given mredGSE (n=3) with and without hydrolysis of glucuronides. Data expressed as means ± SD. Figure S10. Kinetic curve of 4′OMEC in urine of pigs given mredGSE (n=3) with and without hydrolysis of glucuronides. Data expressed as means ± SD. Table S1. Composition of the diet (g/kg) that was fed to pigs (1.5 kg per day in two equal portions) of the control group (n=2) and the group given mredGSE (n = 3). [file mnfr0056-0653-SD1.doc]

Supporting information

**Urinary excretion and metabolism of procyanidins in pigs**

Sebastian Rzeppa^1^, Katharina Bittner^1^, Susanne Döll^2^, Sven Dänicke^2^ and Hans-Ulrich Humpf^1*^

^1^Institute of Food Chemistry, University of Münster, Germany

^2^Institute of Animal Nutrition, Friedrich-Loeffler-Institute (FLI), Federal Research Institute for Animal Health, Braunschweig, Germany

^*^ Corresponding author:

Prof. Dr. Hans-Ulrich Humpf

Westfälische Wilhelms-Universität Münster

Institute of Food Chemistry

Corrensstrasse 45

48149 Münster, Germany

Tel.: +49 251 8333391

Fax: +49 251 8333396

[humpf@uni-muenster.de](mailto:humpf@uni-muenster.de)

**
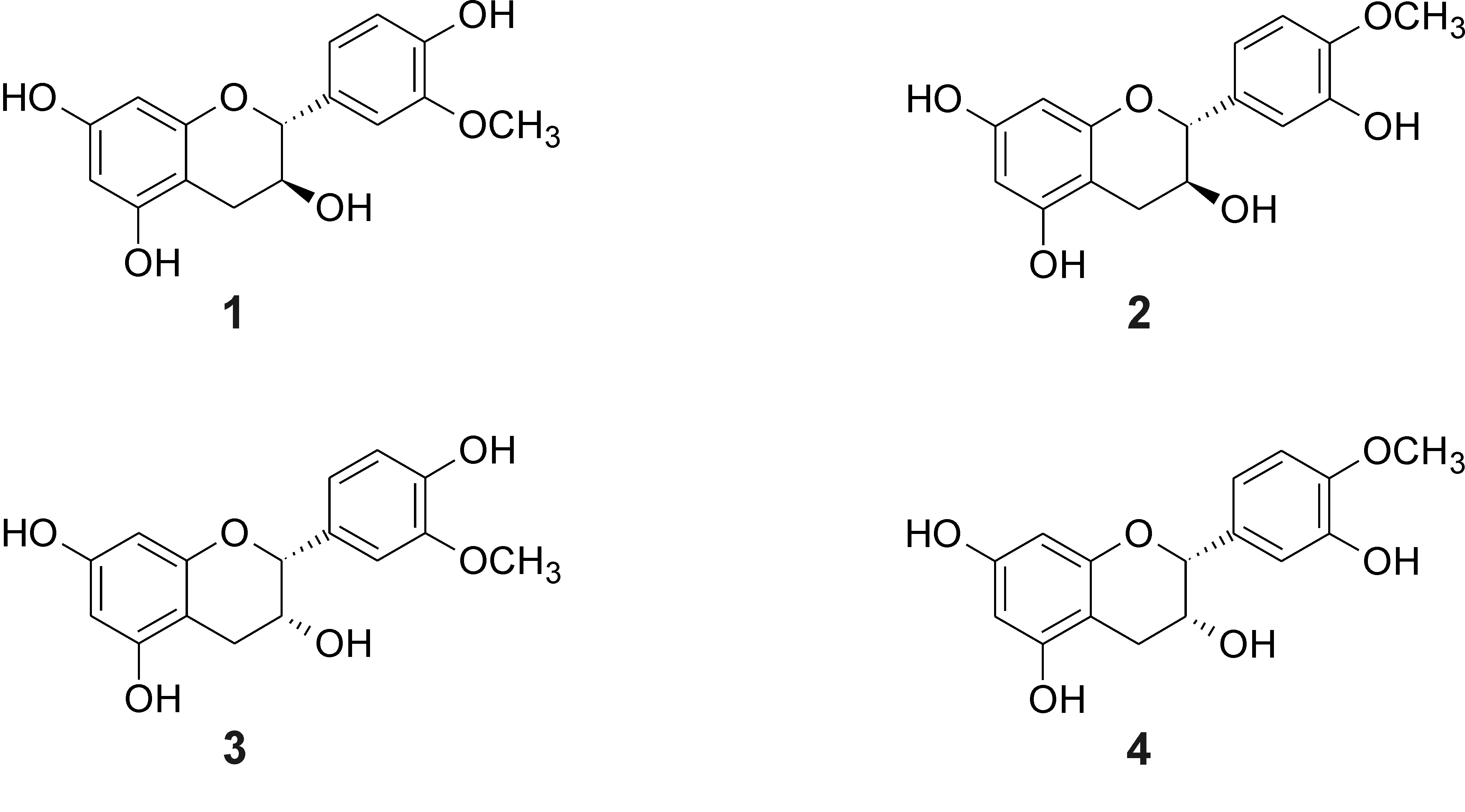
**

**Figure S1.** Structures of methyl derivatives of catechin and epicatechin: 3´-*O*-methyl-(+)-catechin (**1**), 4´-*O*-methyl-(+)-catechin (**2**), 3´-*O*-methyl-(–)-epicatechin (**3**) and 4´-*O*-methyl-(–)-epicatechin (**4**).

**Table S1.** Composition of the diet (g/kg) which was fed to pigs (1.5 kg per day in two equal portions) of the control group (n=2) and the group given mredGSE (n=3). To the feed of the last group 250 mg/kg body weight mredGSE was added in a single dosis at the starting point of kinetic study.


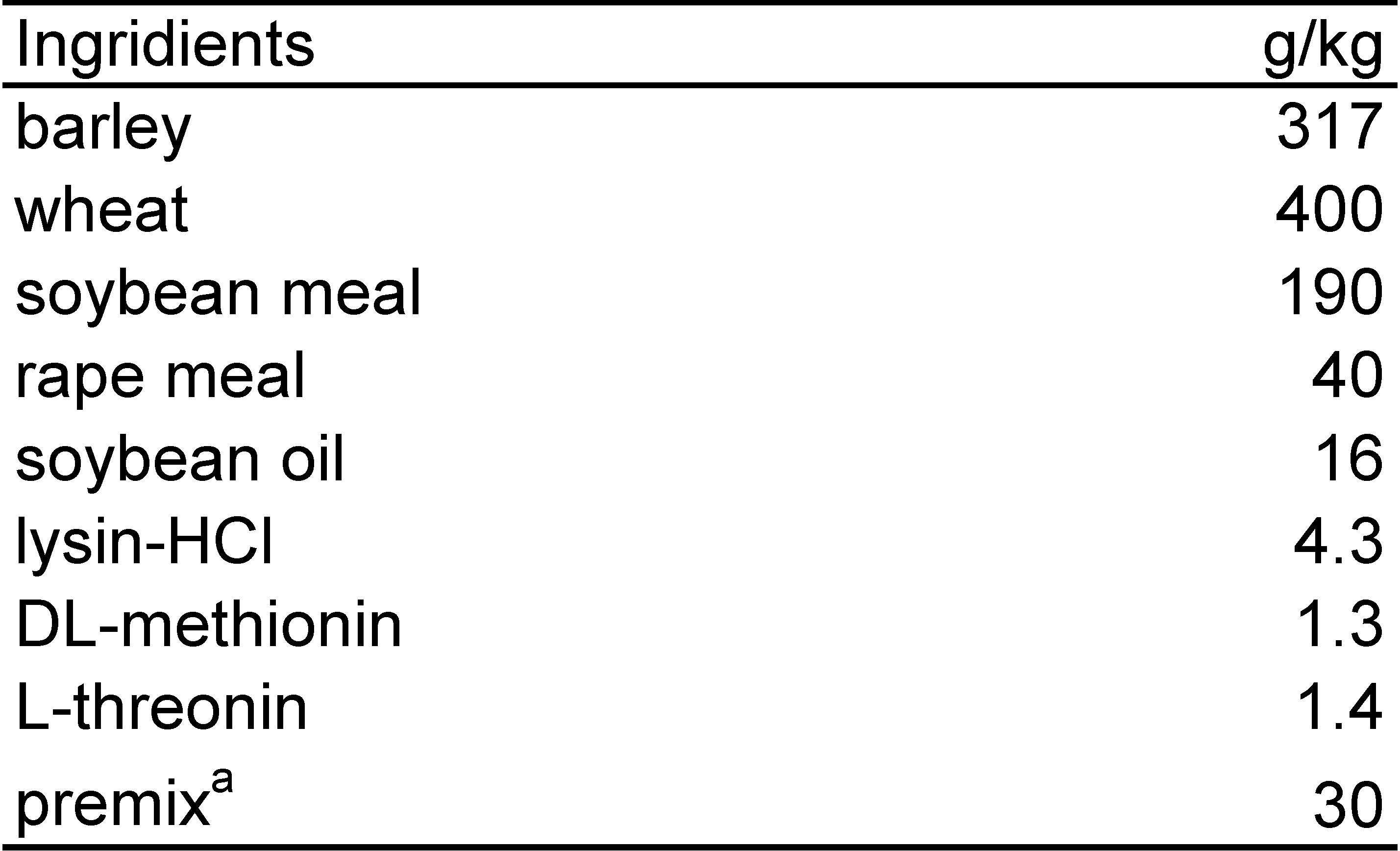


^a^ provided per kg diet: vitamin A 12000 IU; vitamin D_3_ 1200 IU; vitamin E 36 mg; vitamin B_1_ 1.1 mg; vitamin B_2_ 3 mg; vitamin B_6_ 3 mg; vitamin B_12_ 22.5 µg; vitamin K_3_ 1.6 mg; nicotinic acid 15 mg; Ca-panthothenat 10.1 mg; choline chloride 150 mg; Fe 120 mg; Cu 15 mg; Mn 80.1 mg; Zn 100.2 mg; I 2 mg; Se 0.4 mg; Co 0.8 mg.


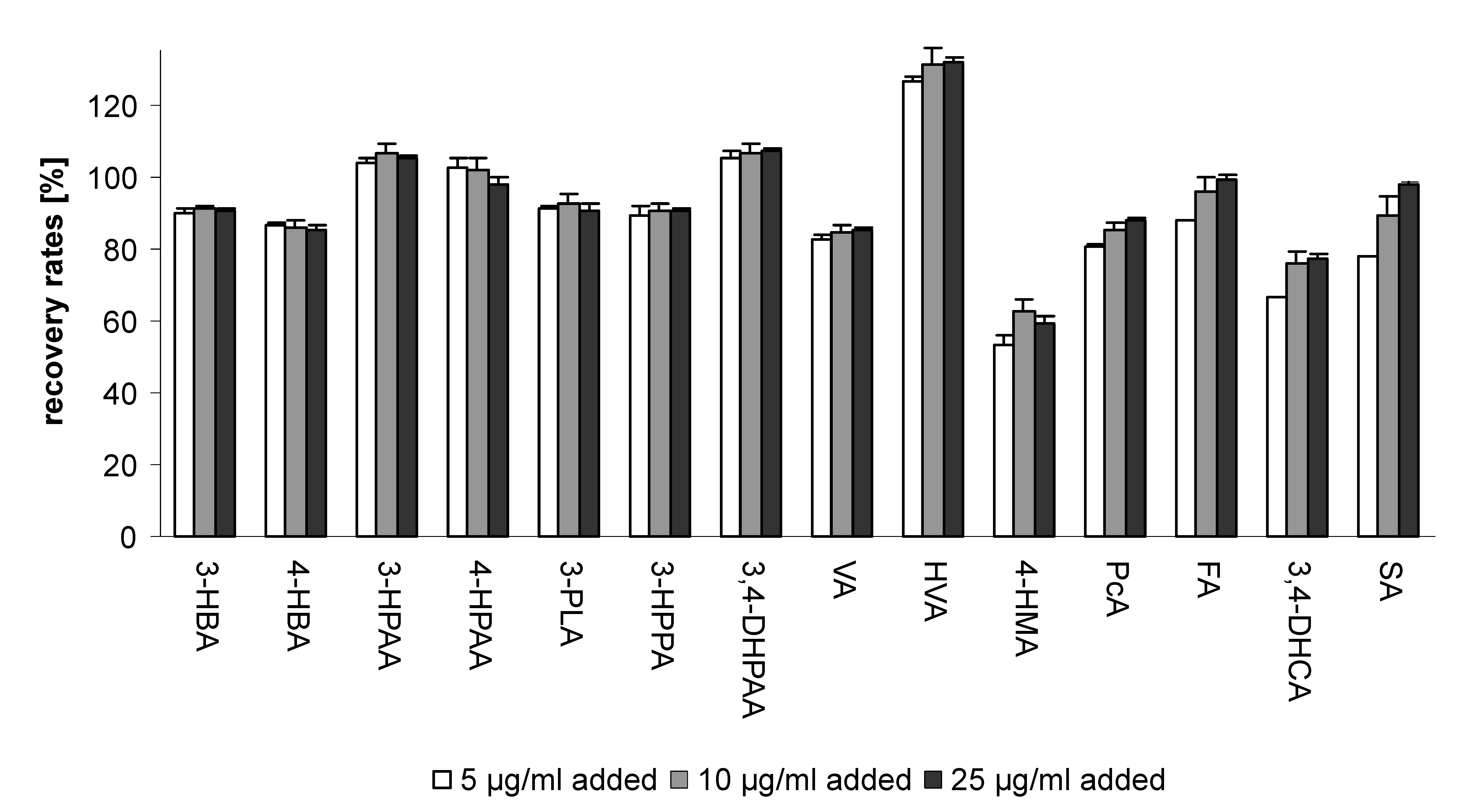


**Figure S2.** Recovery rates [%] of phenolic acids in pig urine after subtraction of naturally occurring concentrations. 3-hydroxybenzoic acid (3-HBA); 4-hydroxybenzoic acid (4-HBA); 3-hydroxyphenylacetic acid (3-HPAA); 4-hydroxyphenylacetic acid (4-HPAA); 3-phenyllactic acid (3-PLA); 3-hydroxyphenylpropionic acid (3-HPPA); 3,4-dihydroxyphenylacetic acid (3,4-DHPAA); vanillic acid (VA); homovanillic acid (HVA); 4-hydroxymandelic acid (4-HMA), protocatechuic acid (PcA); ferulic acid (FA), 3,4-dihydroxycinnamic acid (3,4-DHCA); sinapic acid (SA). Sample clean up by liquid-liquid extraction and measurement by GC-MS. Data expressed as means ± SD (n=3).

**
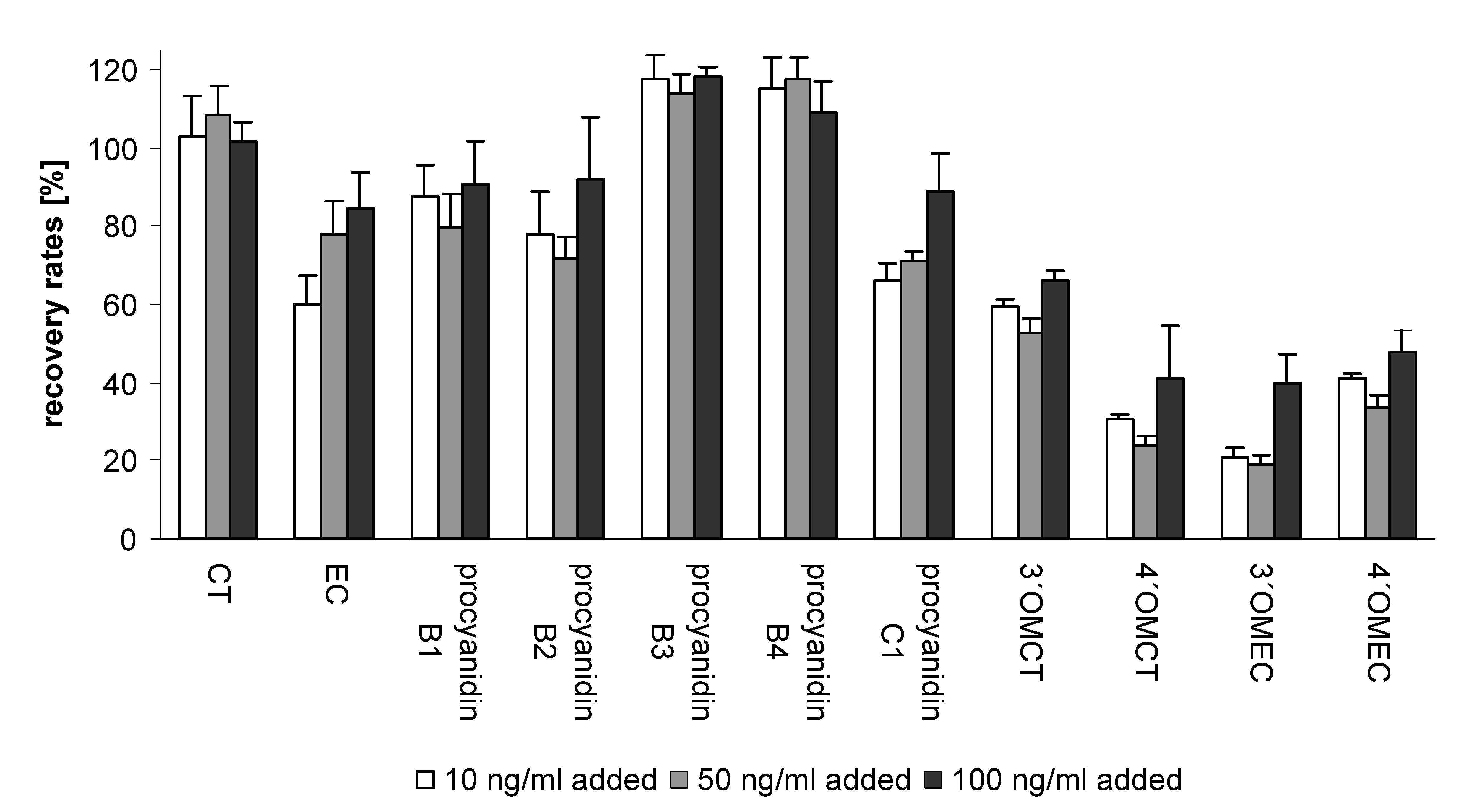
**

**Figure S3.** Recovery rates [%] of flavan-3-ols, methyl derivates of flavan-3-ols and procyanidins in pig urine after subtraction of naturally occurring concentrations. Sample clean up with solid phase extraction with Sephadex LH20 and measurement by HPLC-MS/MS. Data expressed as means ± SD (n=3).


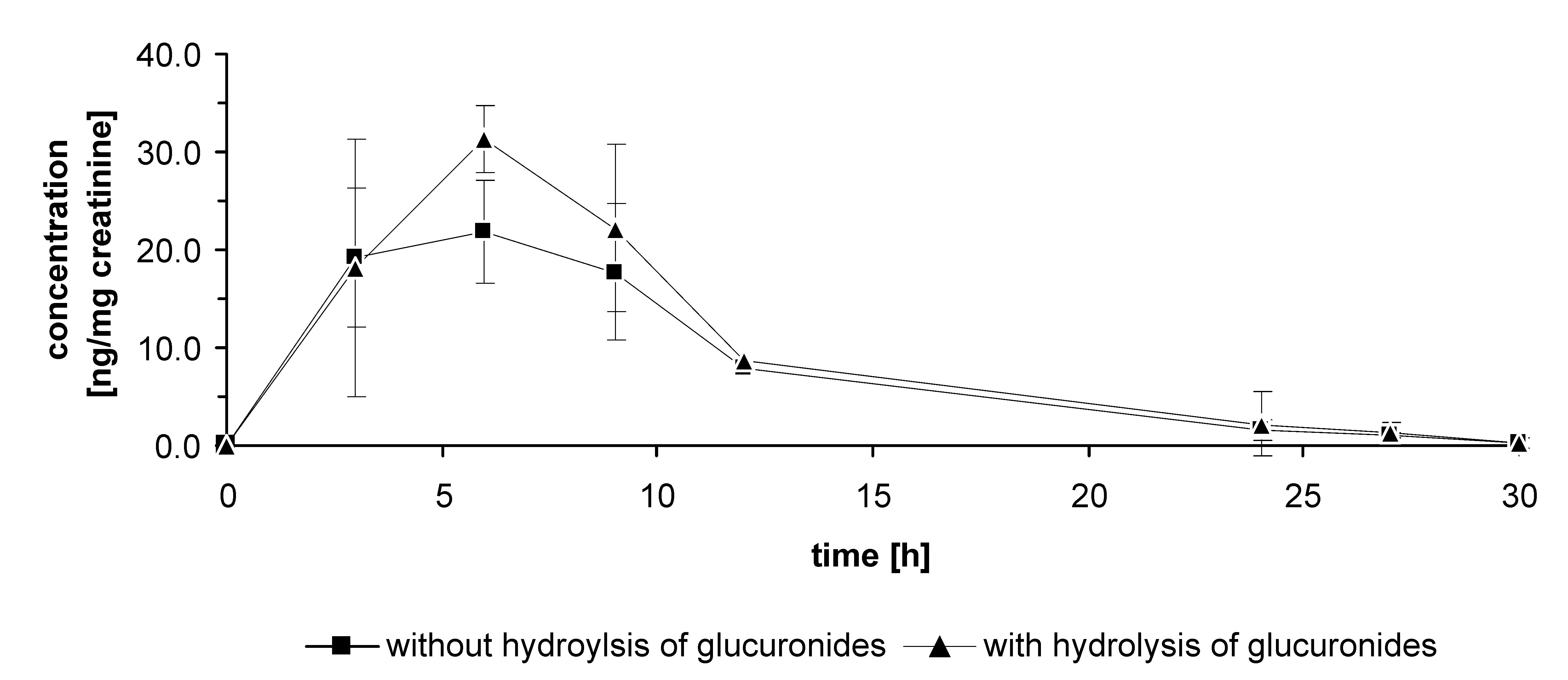


**Figure S4.** Kinetic curve of procyanidin B1 in urine of pigs given mredGSE (n=3) with and without hydrolysis of glucuronides. Data expressed as means ± SD.


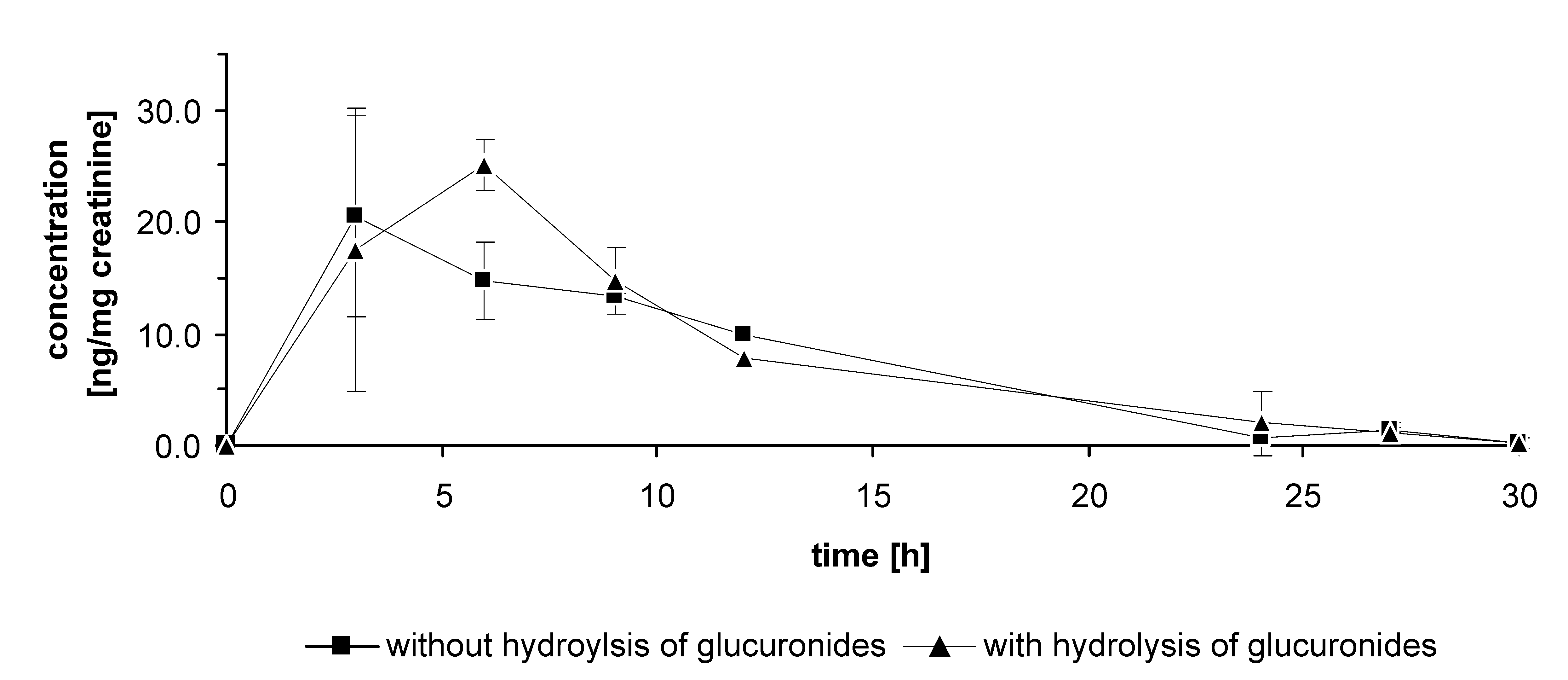


**Figure S5.** Kinetic curve of procyanidin B2 in urine of pigs given mredGSE (n=3) with and without hydrolysis of glucuronides. Data expressed as means ± SD.


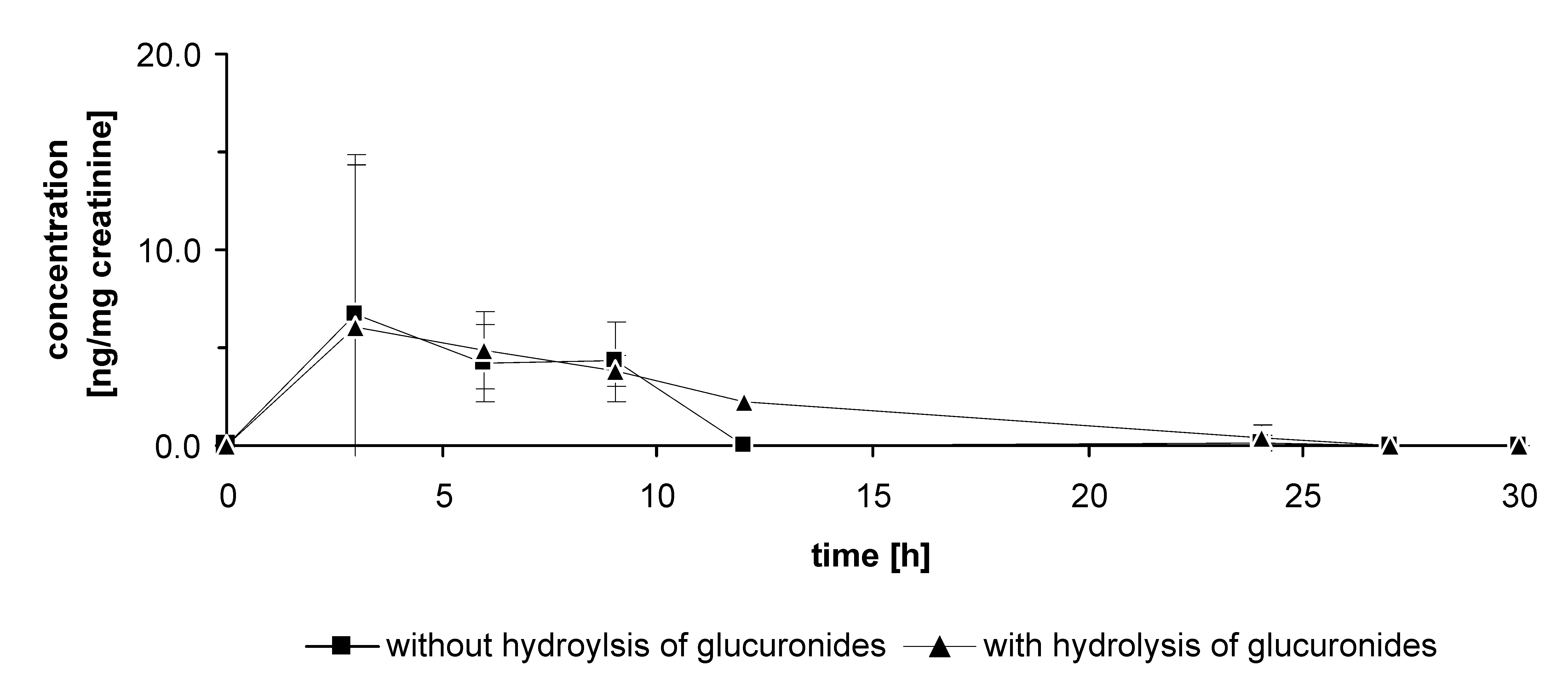


**Figure S6.** Kinetic curve of procyanidin C1 in urine of pigs given mredGSE (n=3) with and without hydrolysis of glucuronides. Data expressed as means ± SD.


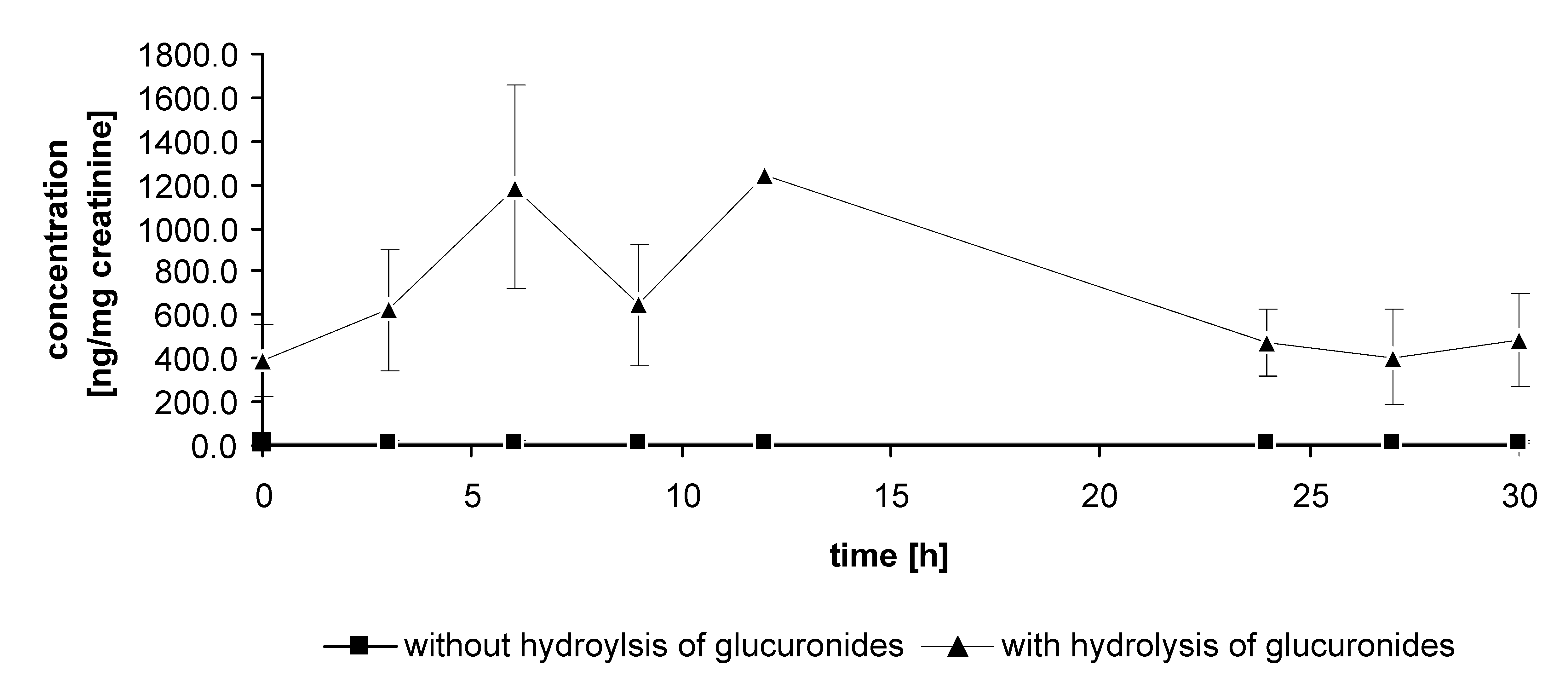


**Figure S7.** Kinetic curve of 3´OMCT in urine of pigs given mredGSE (n=3) with and without hydrolysis of glucuronides. Data expressed as means ± SD.


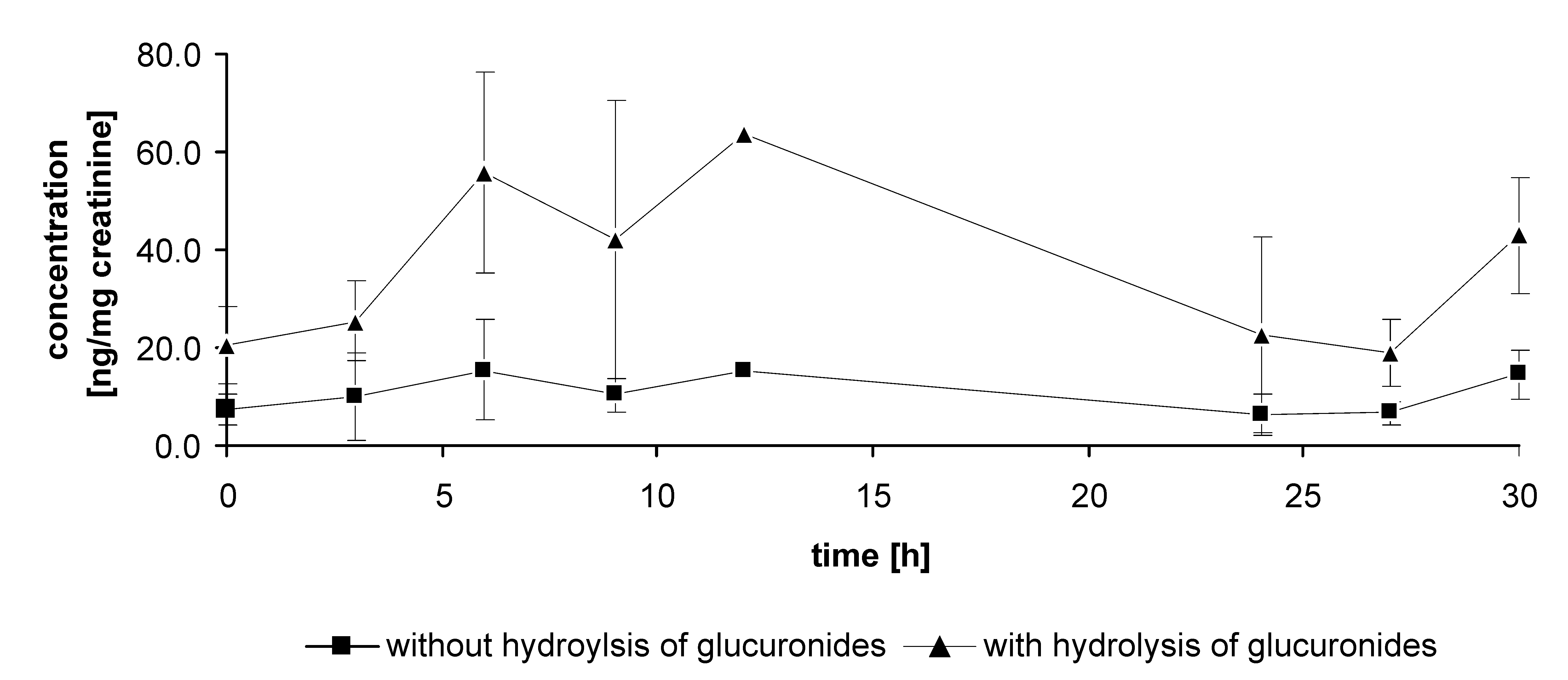


**Figure S8.** Kinetic curve of 4´OMCT in urine of pigs given mredGSE (n=3) with and without hydrolysis of glucuronides. Data expressed as means ± SD.


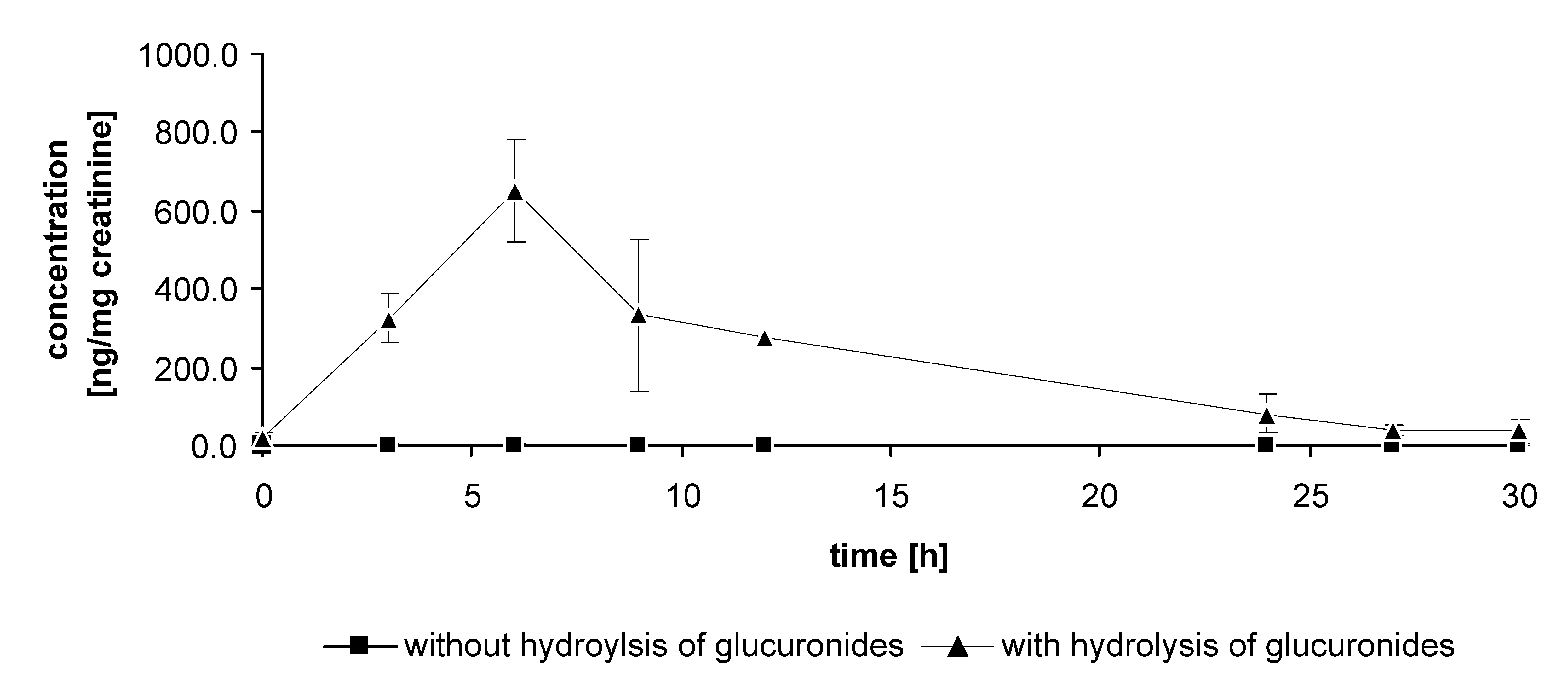


**Figure S9.** Kinetic curve of 3´OMEC in urine of pigs given mredGSE (n=3) with and without hydrolysis of glucuronides. Data expressed as means ± SD.


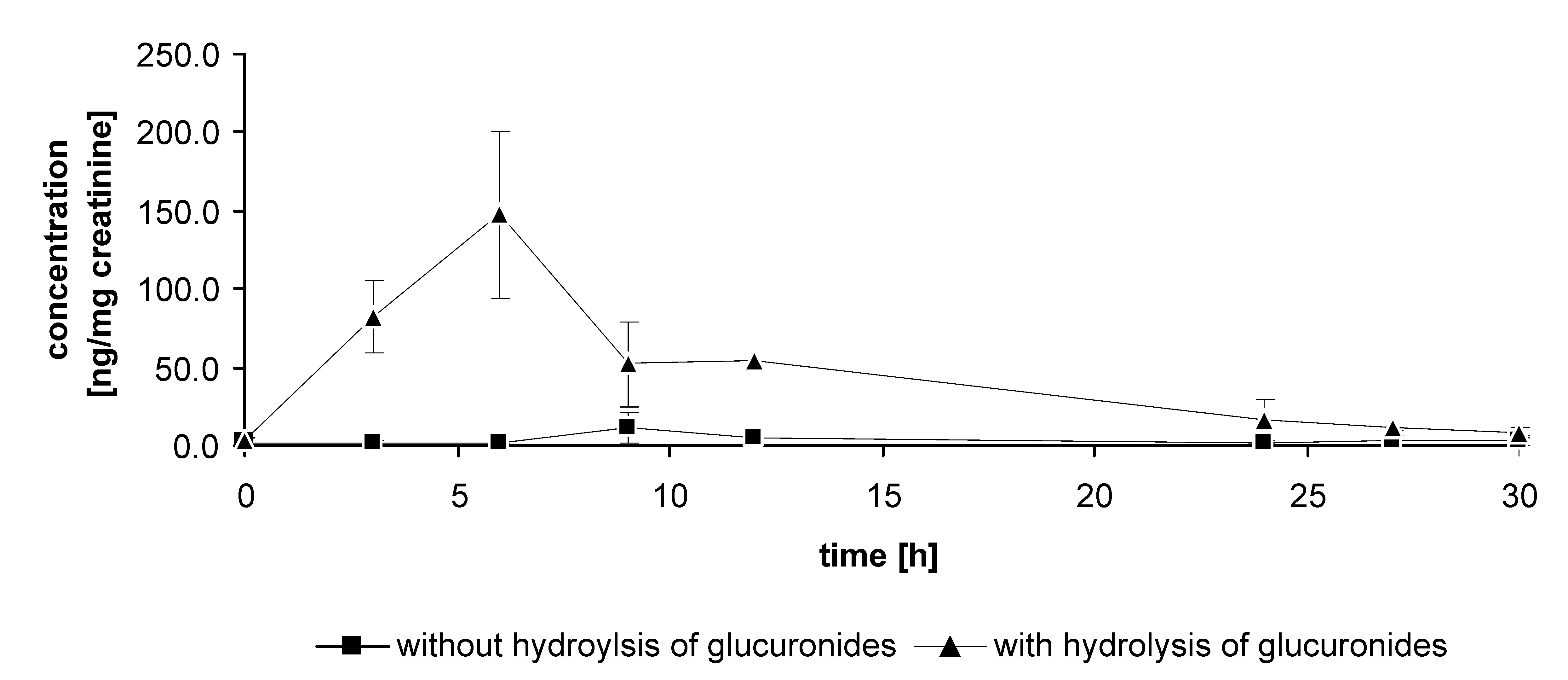


**Figure S10.** Kinetic curve of 4´OMEC in urine of pigs given mredGSE (n=3) with and without hydrolysis of glucuronides. Data expressed as means ± SD


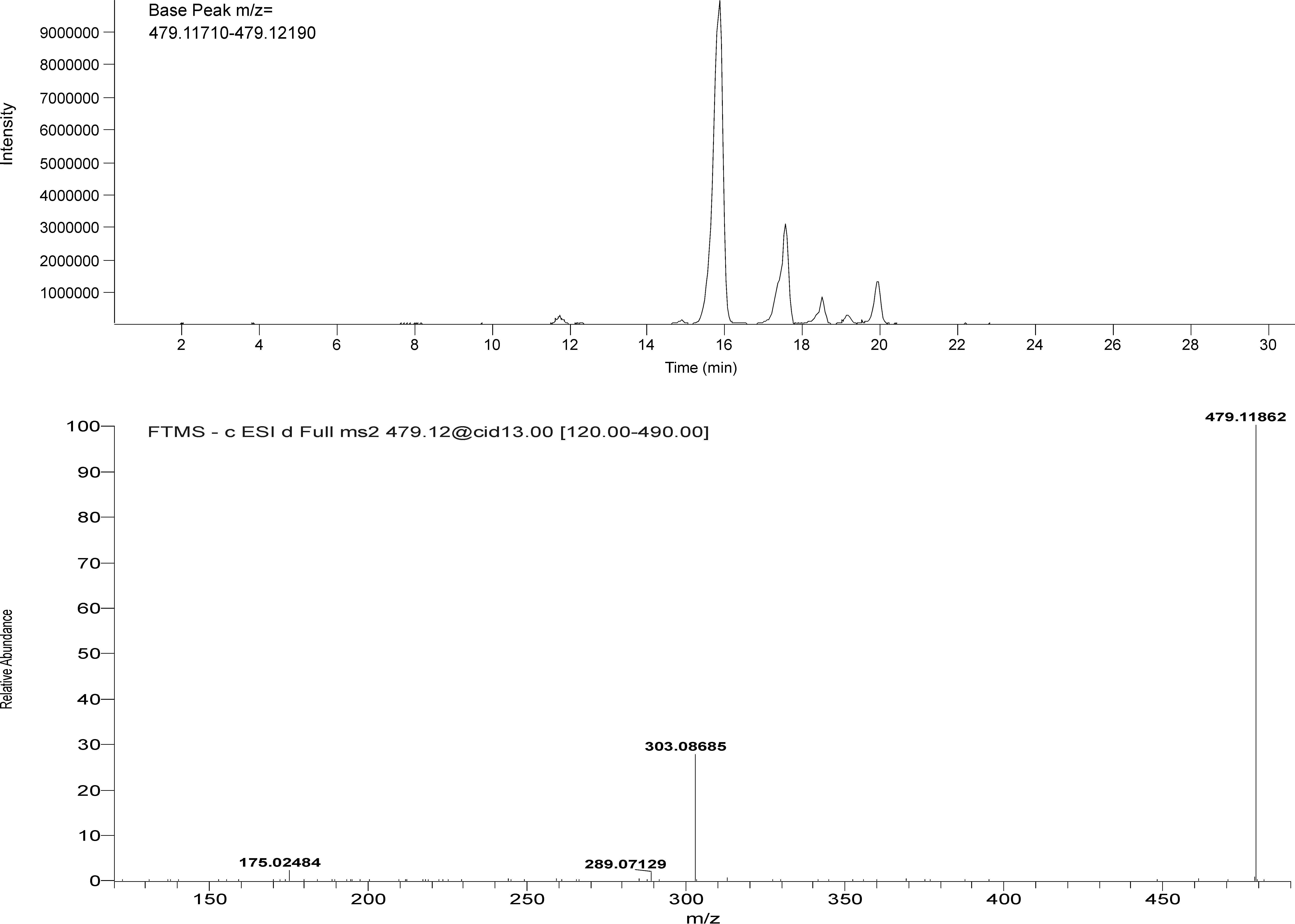


**Figure S11.**: RP-HPLC-FTMS chromatogram of a urine sample of a pig given mredGSE 3 h after starting the kinetic study. Peaks with *m/z* 479.1187 [M-H]^-^ correspond to glucuronides of methylated flavan-3-ols with a calculated mass of *m/z* 479.1195 [M-H]^-^ (above). MS/MS spectrum of the first peak with *m/z* 479.12 [M-H]^-^ with normalized collision energy (CID) 13% (below). Signal with *m/z* 303.0869 [M-H]^-^ corresponds to methyl-flavan-3-ol (calculated mass *m/z* 303.0874 [M-H]^-^) and signal with *m/z* 289.07129 [M-H]^-^ corresponds to non-methylated flavan-3-ol (calculated mass *m/z* 289.0718 [M-H]^-^) (below). Further three peaks show the same fragmentation pattern.
